# Nitriding of 316L Steel in a Glow Discharge Plasma

**DOI:** 10.3390/ma15093081

**Published:** 2022-04-24

**Authors:** Tadeusz Frączek, Rafał Prusak, Marzena Ogórek, Zbigniew Skuza

**Affiliations:** 1 Department of Materials Engineering, Faculty of Production Engineering and Materials Technology, Czestochowa University of Technology, 42-201 Czestochowa, Poland; tadeusz.fraczek@pcz.pl; 2 Department of Production, Faculty of Production Engineering and Materials Technology, Czestochowa University of Technology Management, 42-201 Czestochowa, Poland; rafal.prusak@pcz.pl (R.P.); zbigniew.skuza@pcz.pl (Z.S.)

**Keywords:** austenitic steel, ion nitriding, active screen method, plasma potential

## Abstract

The article presents the results of the research on the nitriding process of 316L austenitic steel and the change in surface properties resulting from this process used in medicine, orthopedics, and in fuel cells. The processes were carried out with the following parameters: time from 5 to 17 h, temperature from 430 °C to 490 °C. The study presents the results of tests of the 316L austenitic steel substrate layer subjected to plasma nitriding of a direct current glow discharge, i.e., in the area isolated from both the cathode and the anode. Additionally, the influence of the active screen on the nitriding process in this area of the direct current discharge was studied. The following tests were carried out: nitrogen diffusion depth test, hardness test, wear resistance test, microstructure analysis, corrosion resistance, and distribution of the element concentration in the surface layer. The research allowed for the conclusion that each variant of nitriding contributed to a change in the examined properties, while the observed scale and nature of the changes were different.

## 1. Introduction

Austenitic steels are non-magnetic stainless steels that contain high chromium and nickel content and low carbon content in their chemical composition. These steels have a number of desirable properties, such as corrosion resistance, good molding properties, and good weldability. Good corrosion resistance results from the presence of an oxide layer on the passive surface, consisting mainly of chromium oxide, which regenerates itself in case of damage and protects the alloy from environmental influences [1,2]. Low hardness and poor tribological properties may have a negative impact on the operation of components exposed to abrasive wear, which may limit the industrial use of austenitic steels in some areas [3,4]. Moreover, these alloys are subject to local corrosion, especially pitting corrosion, in specific environments, especially in solutions containing chloride ions. An important technique that allows for improving corrosion properties and abrasion is low-temperature nitriding [5,6,7]. Traditional nitriding of austenitic stainless steels at temperatures above 500 °C leads to the precipitation of CrN, which causes a significant decrease in corrosion resistance [8,9]. Ion nitriding of austenitic steel with the active screen method offers many advantages over the conventional cathode ion nitriding method under direct current glow discharge conditions, such as better surface quality and layer homogeneity [10], and it has been used, e.g., in medicine [11,12], orthopedics [13], and in fuel cells [14]. The basic features characterizing ion nitriding are [15]:

- A process carried out in an atmosphere of N_2_ + H_2_,
- as a result of diffusion, a layer of nitrides and a solid nitrogen solution in austenite is created,
- raising the surface temperature of the material resulting from the collisions of the ions,
- the duration of the process can be reduced by using an active plasma containing nitrogen atoms and ions.

This method leads—at a low temperature—to the diffusion of nitrogen atoms on the material surface and the formation of a metastable layer of nitrides and a supersaturated austenitic γN phase [16]. The formation of this phase contributes to increasing the hardness of the steel while not reducing the corrosive properties. The positive effect of low-temperature ion nitriding can be seen, among others, in 316L and 304 [17].

The research results presented in the literature on the subject focus on the analysis of various aspects of the nitriding process of austenitic steels. The tests carried out for 316L steel showed that the formation of a thin layer of austenite supersaturated with nitrogen does not affect the mechanical properties and the γ-α phase transformations under low-temperature deformation conditions [18]. The conducted research aimed at determining the influence of the composition of the gas mixture on the nitriding process allowed us to conclude that the increase in the N_2_^+^ content in the plasma contributes to the increase in the thickness of the nitride layer and the increase in the solid nitrogen content. At the same time, the use of Ar-N_2_ plasma causes inconsiderable surface penetration by nitrogen [16]. Research by Borgioli et al. [5] enabled us to conclude that nitrides were formed during nitriding at the temperature of 430 °C, and their amount was influenced by the composition of the steel. Nitriding treatments enabled a significant increase in surface microhardness and corrosion resistance in comparison to untreated alloys. In the absence of nitrides, as in the case of nitrided samples at the temperature of 400 °C, the corrosion resistance of the considered steels was comparable. The formation of nitrides on the surface of the zones negatively affected the corrosion resistance, intensifying the corrosion reactions. The precipitation of nitrides affected the corrosion resistance, intensifying the corrosion reactions. At the same time, tests carried out by Zhang and Bell [19] at a temperature of 570 °C under various process conditions, allowed to state that—due to the presence of γ′ nitride—the two-layer surface of the phase compound has better corrosion properties in relation to ion-nitrided stainless steel γ′ and austenite. Moreover, low-temperature plasma nitriding at 400 °C made it possible to create a nitrided layer, the corrosion resistance of which was comparable to that of the base material. The studies of other authors allowed to state that the highest surface hardness was obtained after nitriding at the temperature of 460 °C while obtaining layers with a thickness of 10 µm. The conducted X-ray diffraction studies showed that the CrN nitride phase was formed, and underneath it there was a zone of austenite supersaturated with nitrogen [17].

This study presents the results of tests of the 316L austenitic steel substrate layer subjected to plasma nitriding of a direct current glow discharge, i.e., in the area isolated from both the cathode and the anode. Additionally, the influence of the active screen on the nitriding process in this area of the direct current discharge was studied. The obtained test results were compared with the results of cathode and cathode + active screen nitriding presented earlier in the paper [20].

## 2. Materials and Research Methodology

The material used in the tests, the results of which are included in this article, was 316L austenitic steel (Table 1). The paper analyzes the results for various variants of the location of the samples subjected to ion nitriding in the working chamber of the furnace:

- in the plasma potential—on the surface insulated both from the anode and from the cathode,
- in the plasma potential with the use of an additional active screen.

After conducting the ion nitriding processes, the analysis of the test results also considered two other variants of the sample arrangement (without the use of plasma): directly on the cathode and on the cathode with the use of an active screen [20].

Chemical composition in accordance with the certificate no. MEST 451139/2007.

Cylindrical samples with dimensions of Ø22 × 7 mm were subjected to nitriding. The surfaces of the samples intended for glow discharge nitriding were ground with sandpaper with a grade of 2500. Immediately before loading into the glow chamber, the samples were degreased with acetone. In the initial stage of the process, the samples were subjected to ion bombardment in an argon-hydrogen atmosphere (20–80%) in order to remove the passive layer (Cr_2_O_3_), preventing the nitriding process.

**Figure 1:**
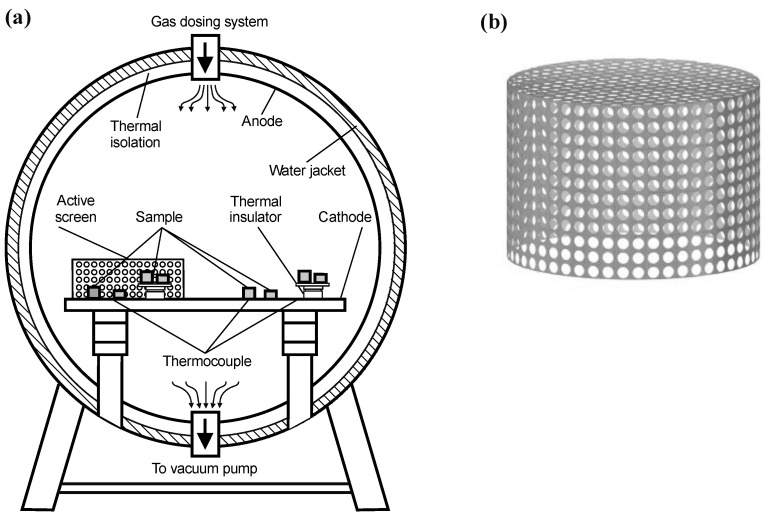
Diagram of a modified glow discharge nitriding device with a visible active screen: (**a**) diagram of the JON-600 furnace, (**b**) active screen.

A modified device with cooled JON-600 anode was used in the tests. The changes introduced in the device construction were related to the development of a modified technology of glow discharge nitriding, combining the advantages of the device with a cooled anode with the simultaneous intensification of surface processes. Glow discharge nitriding technology—with the use of an active screen—intensifying surface processes was used (Figure 1a), as a result of which the depth of the nitrided layer on glow discharge nitrided metallic materials is increased. A cylindrical active screen made from perforated sheet metal with a chemical composition similar to the material of the nitrided elements (Figure 1b). The basic features of the active screen used were as follows:

- shape: cylinder without bottom plane,
- dimensions: diameter 200 mm, height 100 mm,
- hole size: diameter 5 mm,
- 5 mm hole spacing.

A modified glow discharge nitriding device has been prepared to form nitrogen layers obtained after nitriding the elements placed on the cathode, as well as on the surface insulated from the cathode and anode (the so-called “plasma potential”), by using a ceramic insulator with an internal labyrinth. Location of the nitrided element on the insulator or cathode—determines its temperature value.

The processes of basic glow discharge nitriding were conducted in a device for glow discharge treatments, placing the nitrided elements in the so-called “plasma potential”—an area isolated from the cathode and anode. Some of the elements placed in this way were additionally covered with an active screen (Figure 1b).

In the conducted research, process parameters were used in accordance with the rotatable model of experimental planning (Table 2).

In order to assess the quality of the produced nitrided layers, the following tests were conducted:

- Nitrogen diffusion depth and element distribution—the depth of nitrogen diffusion was determined on the basis of elemental arrangement analysis on an optical emission spectrometer with glow discharge (GDEOS GD Profiler HR with a Grimma discharge lamp with a 4 mm cathode diameter).
- Microstructure—observation of the microstructures was performed with an Axiovert microscope with digital image recording.
- Corrosion resistance—AMEL potentiostat, environment of 0.5 mol. NaCl aqueous solution.
- Hardness—microhardness measurements of the nitrided layers were conducted using the Knoop method on a Future Tech FM7 microhardness tester.
- Abrasive wear resistance—the microhardness was measured using 490.3 mN load.

## 3. Tests Results

### 3.1. Test of the Nitrogen Diffusion Depth and Element Distribution

Table 3 presents the results of measurements of the depth of layers nitrided in the plasma potential on the substrate of 316L steel. The obtained results enable us to conclude that the use of an active screen significantly increases the depth of nitrogen diffusion with regard to nitriding only in the plasma potential—that is, in the area insulated from both the cathode and the anode (in the range of 592% to 1015%).

When comparing the obtained results to the parallel tests without using the plasma potential (i.e.,—cathode and cathode + active screen [20]), it should be stated that the greatest depths of nitriding were obtained in the variant with the use of the cathode and active screen (increase in the depth of the layers greater by 9% to 72% in relation to the plasma + active screen variant).

**Figure 2:**
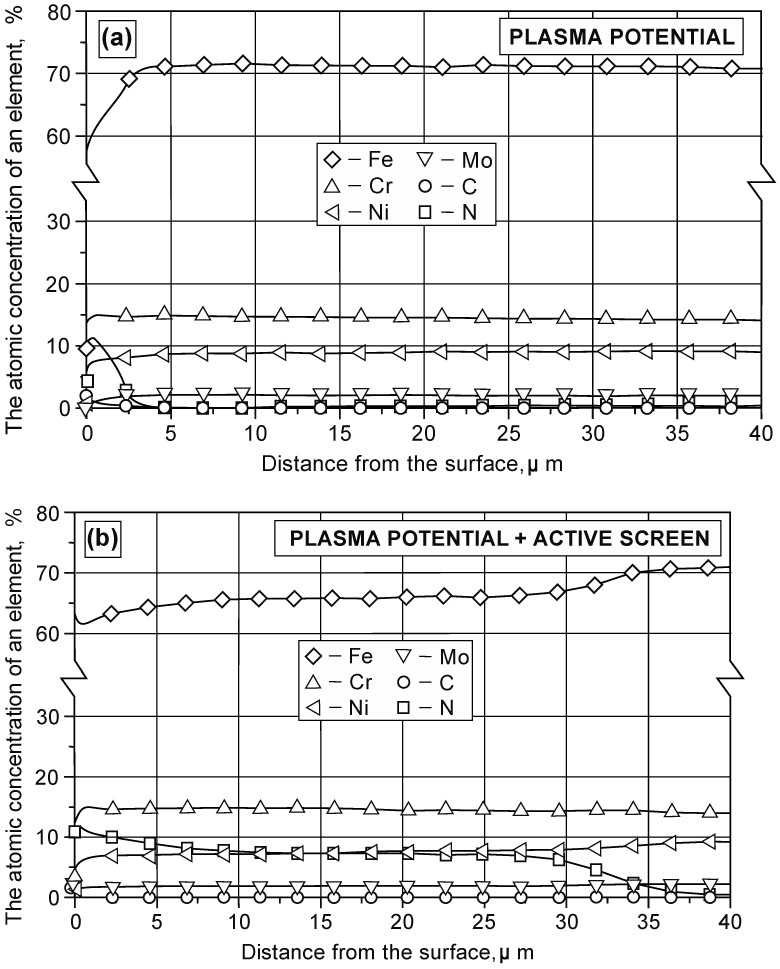
Distribution of the element concentration in the surface layer on the austenitic 316L steel substrate depending on the location in the glow chamber. Temperature T = 460 °C, time t = 11 h: (**a**) plasma potential, (**b**) plasma potential + active screen.

Figure 2 shows the results of the analysis of the distribution of the element concentration in the surface layer on the austenitic 316L steel substrate.

The analysis of nitrogen concentration in the nitrided layer shows that the proportion of chromium and nitrogen concentrations in the near-surface zone is 1:1 (Table 4). This proves the presence of CrN nitrides on the surface of the precipitation zone. The concentration of nitrogen at a distance of a few micrometers from the surface of the layer stabilizes at a certain value level. When taking into account the proportion of the concentration of chromium and nitrogen atoms, its value was determined—approximately 2:1. On this basis, it can be concluded that the next zone of the nitrided layer consists of Cr_2_N nitrides in the austenite matrix.

While analyzing the studied areas of direct current glow discharge, it should be stated that ion nitriding of the austenitic 316L steel elements located on the cathode cause that on the surface of the produced layer there is an area with a high concentration of chromium and nitrogen, compared to areas at a greater depth. Nitriding of elements located in the area insulated from both the anode and the cathode results in a lower concentration of chromium atoms on the surface. The analysis of the structure of the layers formed during nitriding in the plasma potential with the use of an active screen shows that compared to nitriding at the cathode, the depth of the CrN nitride zone decreases, while the width of the zone of the austenite supersaturated with nitrogen γ_N_ increases. The analysis of the linear distribution of the concentration of elements in the nitrided layer on the 316L steel substrate showed that the use of an active screen increases the depth of nitrogen diffusion [21,22]. The active screen during nitriding on the 316L steel cathode made it possible to increase the depth of diffusion from 2 to 4 times, depending on the processing time [20], while in the plasma potential, it increases from 7 to 11 times (Table 3). It should be emphasized that the diffusion layers of the smallest depth formed on the 316L steel substrate also increase the functional properties of nitrided structural elements and machine parts during nitriding in the plasma potential.

**Figure 3:**
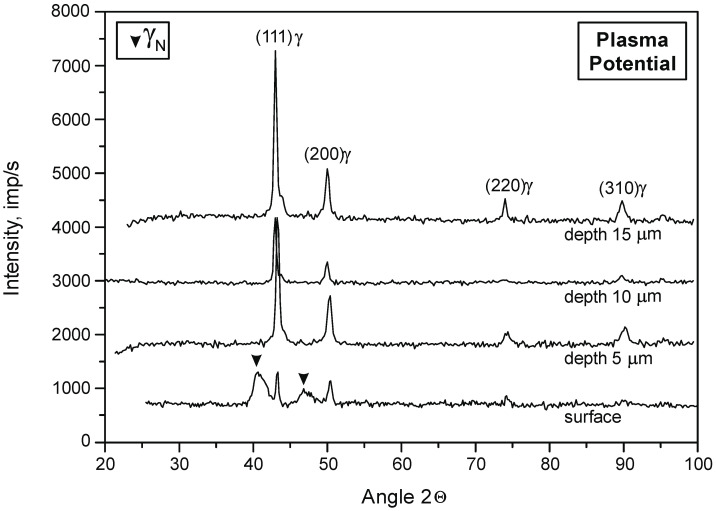
Diffractograms of the nitrided layer on the substrate of 316L steel depending on the depth in the cross-section of the surface layer. Nitriding in plasma potential, T = 490 °C, t = 8 h.

In order to determine the phase composition of the nitrided layer, diffractograms were prepared. The tests were carried out at depths from 5 to 30 µm with a step of 5 µm. The obtained results are consistent with the previous analysis of the chemical composition (Table 4). These results also served as models for the structure of nitrided layers on the 316L steel substrate that were developed for various variants of the location of elements in the glow chamber (Figure 3). A characteristic feature of these layers is the zonal structure—composed of CrN and Cr_2_N nitrides and γ_N_ phase grains. In the microstructure of nitrided layers in the plasma potential, no nitride zones were found (these zones occur both in the plasma + active screen variant and in the cathode and cathode + active screen variants). The width of individual zones changes along with the change of the nitriding variant and the nitriding time.

The results show that the use of an active screen contributes to the intensification of surface processes, as a result of which the depth of the nitrided layer increases and the functional properties of the nitrided elements improve. An active screen increases the concentration of active plasma components inside it, which, combined with appropriate temperature values, affects the kinetics of the process.

**Figure 4:**
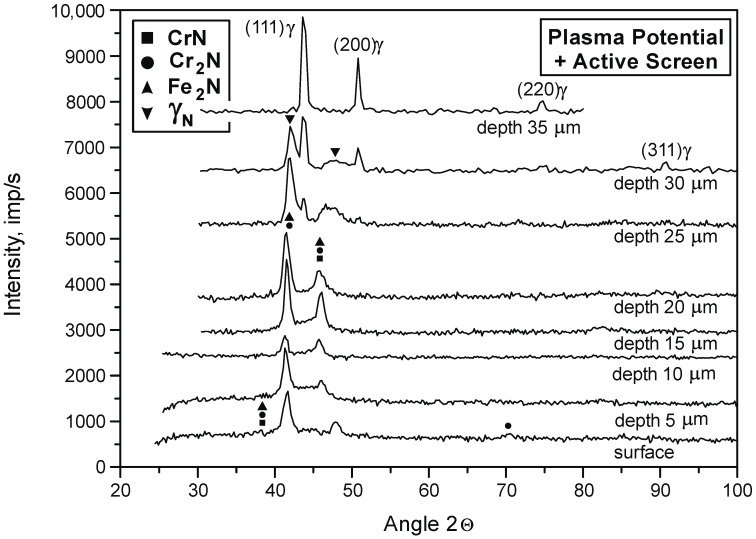
Diffractograms of the nitrided layer on the substrate of 316L steel depending on the depth in the cross-section of the surface layer. Nitriding in plasma potential + active screen, T = 490 °C, t = 8 h.

**Figure 5:**
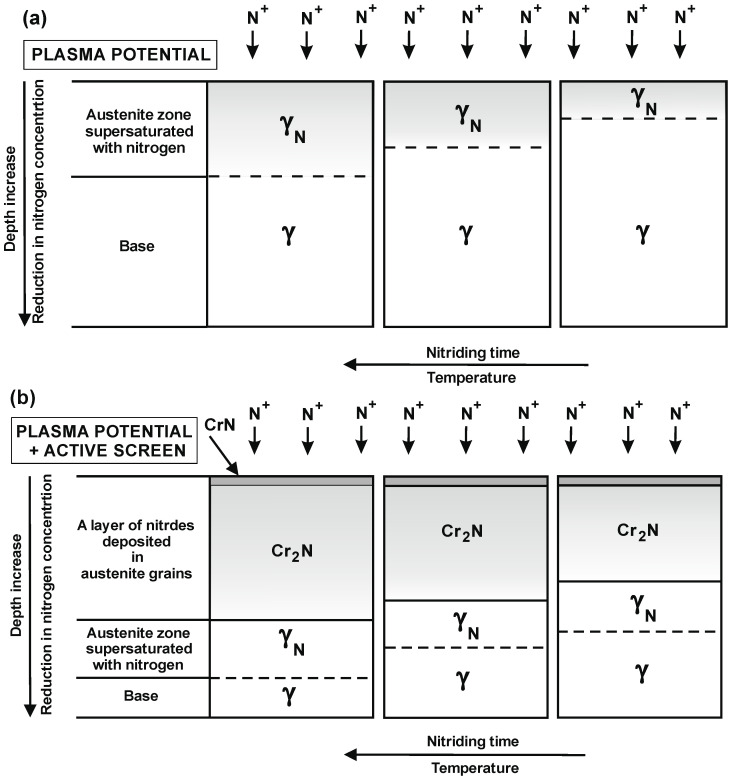
Models of the structure of surface layers on the 316L steel substrate depending on the location in the glow chamber: (**a**) plasma potential, (**b**) plasma potential + active screen.

Figure 4 and Figure 5 show the diffractograms of the nitrided layer on the 316L steel substrate in the plasma potential and the plasma potential with an active screen.

### 3.2. Test of Microstructures

**Figure 6:**
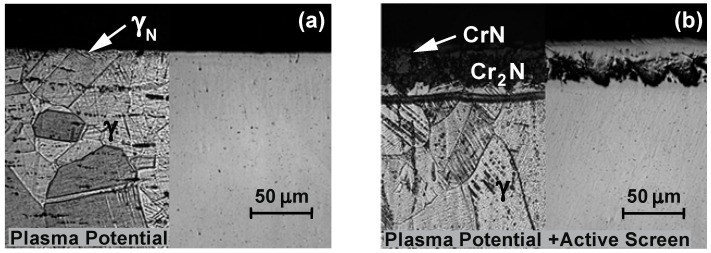
Microstructure of nitrided layers on the 316L steel substrate, temperature T = 490 °C, time t = 14 h; (**a**) plasma potential, (**b**) plasma potential + active screen.

Figure 6 shows the results of the research on the microstructures of the layers nitrided on the 316L steel substrate. It was concluded that during nitriding only in the plasma potential, the depth of the diffusion layer changes—with the time and temperature of nitriding—due to the increase in the thickness of the zone of the austenite supersaturated with nitrogen, i.e., the γ_N_ phase grains. The use of an active screen enables us to conclude that—taking into account the effect of the nitriding time—the total depth of the nitrided layers changes mainly as a result of increasing the depth of the Cr_2_N nitride precipitation zone.

To sum up, it should be stated that nitriding in the plasma potential—due to the lower concentration of chromium atoms on the surface—is not conducive to the formation of a nitride zone. In this variant of nitriding, only the zone of the austenite supersaturated with nitrogen is formed (exponent austenite)—γ_N_. The additional use of nitriding of the active screen for this variant causes both the formation of CrN and Cr_2_N nitride zones, as well as increases the width of the zone of the austenite supersaturated with nitrogen—γ_N_.

The obtained microscopic tests of the 316L steel substrate layers after nitriding (Figure 6, Table 4) correlate well with the results of the chemical composition analysis on their cross-section—using the GDEOS method. It was found that the width of individual zones depends on the time and temperature as well as the position of the element in the glow chamber during nitriding.

**Figure 7:**
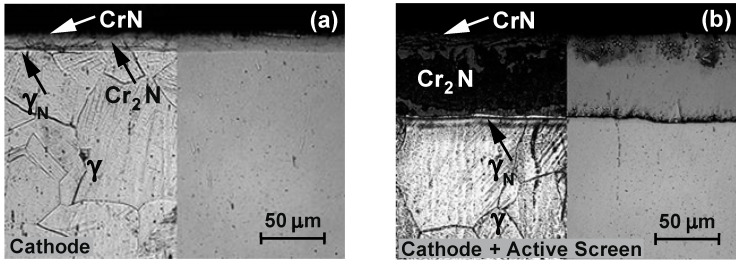
Microstructure of nitrided layers on the 316L steel substrate, temperature T = 490 °C, time t = 14 h; (**a**) cathode, (**b**) cathode + active screen.

When relating the obtained test results in the plasma potential to the nitriding processes at the cathode and cathode with the use of an active screen (Figure 6 and Figure 7), it should be stated that during the nitriding on the cathode with the use of an active screen, the largest zone of precipitation of Cr_2_N nitrides is formed.

### 3.3. Test of Hardness

The analysis of the results of hardness measurements of the obtained surface layers and on the profile of their cross-section—using the Knopp method—showed that their hardness increases for all the adopted conditions of the nitriding process (Table 4). It should be stated that the layers after glow discharge nitriding in the plasma potential have a lower hardness than the layers produced under the conditions of the nitriding process with the use of an active screen and on the cathode [20] (the highest hardness was obtained with cathode nitriding with an active screen)—the percentage increase for the cathode + active screen variant compared to the initial state was from 315% to 409%; the percentage increase in the hardness of the cathode + active screen variant to the value of the plasma potential + active screen was from 19% to 39%.

The process of nitriding this steel in the plasma potential leads to the formation of a diffusion layer of the smallest depth and hardness. Increasing the temperature and increasing the nitriding time cause a significant increase in the hardness of the tested austenitic steel (Table 5).

### 3.4. Tribological Research

The abrasion resistance test and the determination of the friction coefficient value were carried out on a T-05 tribological tester (sample dimensions: 4 × 8 × 12 mm, 100Cr6 steel roll, hardness 62 HRC: diameter 35 mm). The value of the friction coefficient *μ* was determined through the formula:

$$\mu =\frac{T}{F_{N}}$$

where: *T*—force of friction, *F_N_*—load force.

**Figure 8:**
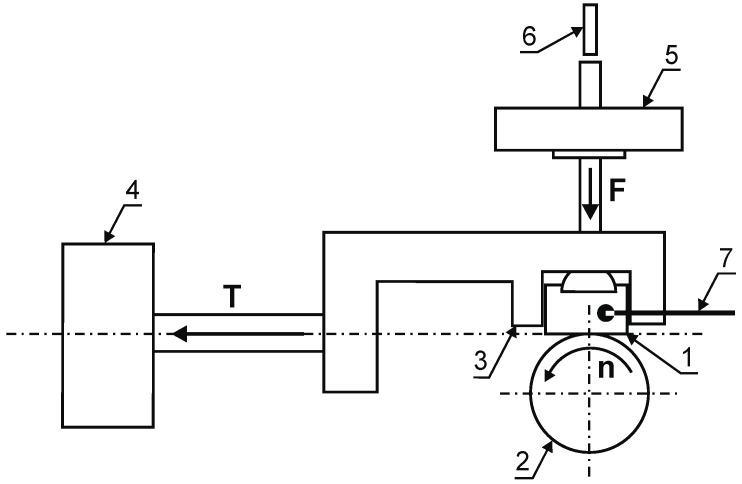
The diagram of a T-05 tester: (1) nitrided layer, (2) roll, (3) sample holder, (4) sensor for measuring the friction force, (5) load, (6) displacement sensor, (7) thermocouple.

The value of the friction coefficient for the austenitic 316L steel was determined for a load of 8.83 N and a time of 7 min. The analysis of the obtained friction coefficients of the layers produced on the 316L steel substrate shows that they are close to the value of the coefficient obtained for this steel before the nitriding process. An abrasive wear resistance test was conducted for a load of 23.54 N, a duration of 1 h, and a linear velocity of 1 m/s (friction path of 10.8 km). The diagram of the tribological tester is presented in Figure 8.

Glow discharge nitriding of the austenitic 316L steel substrate increased its abrasive wear resistance for each nitriding process (Table 6). The smallest increase in wear resistance was observed for the nitrided layers in the glow discharge plasma potential, especially for a short time and at low temperatures of the process. It was found that the use of such nitriding process conditions made it possible to double the abrasive wear resistance.

For the plasma + active screen variant, a maximum nearly 240-fold increase in abrasive wear resistance was found in relation to nitriding in the plasma potential. However, it should be emphasized that the largest 900-fold increase in abrasive wear resistance was obtained for layers produced during cathodic nitriding with an active screen and a processing time of 17 h.

Research has shown that extending the time and increasing the temperature of the process, resulting in an increase in the diffusion speed, contributes to the improvement of tribological properties.

### 3.5. Test of Corrosion Resistance

The corrosion resistance tests of the formed nitrided layers on the austenitic 316L steel substrate were conducted on a test stand in the environment of 0.5 mol. of NaCl aqueous solution. The potentiodynamic method (AMEL 7050 potentiostat) was used (the reference electrode was a calomel electrode). Contamination was removed from the surface layers of nitrided samples in ultrasonic cleaning. The measurements of the electric potential were carried out accurately to within 1 mV/s.

**Figure 9:**
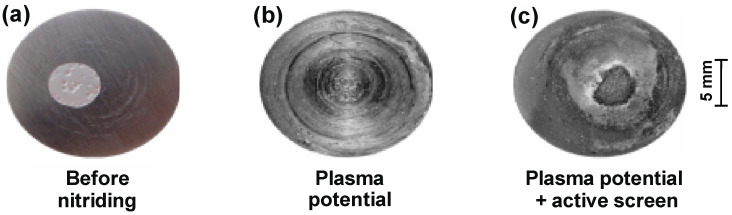
Image of nitrided 316L steel substrate, after potentiodynamic tests: (**a**) before nitriding, (**b**) plasma potential, (**c**) plasma potential + active screen.

The conducted tests enabled us to conclude that the nitriding of the austenitic 316L steel in the area of the glow discharge plasma leads to an increase in the resistance of the nitrided layer to pitting corrosion (Figure 9) with a simultaneous deterioration of the stability of the passive layer.

**Figure 10:**
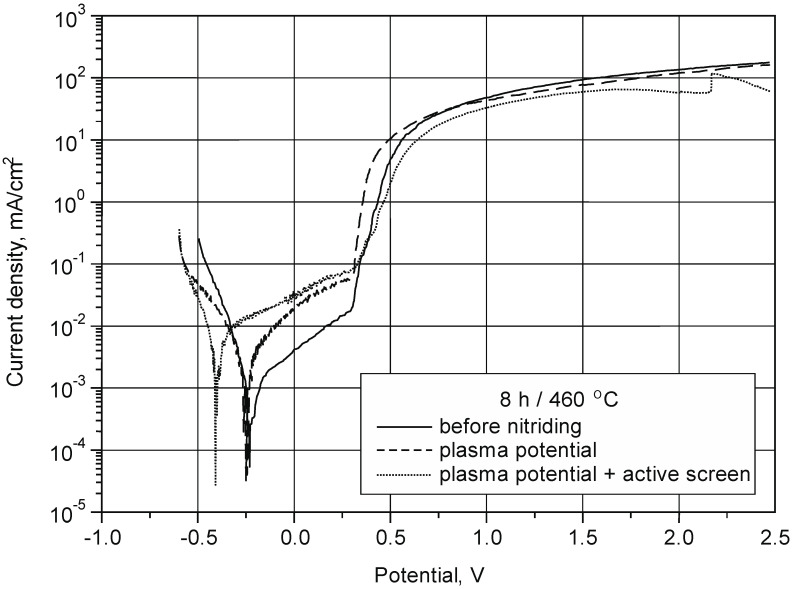
Potentiodynamic curves of the tested layers of 316L steel before and after nitriding in different variants.

Potentiodynamic curves of nitrided layers on the austenitic 316L steel substrate are marked by the absence of active dissolution peaks (The nitrided layer on the substrate of this steel undergoes spontaneous passivation in the applied environment. The current density in the passive range has the lowest value for a non-nitrided surface (10^−3^¸10^−2^ mA/cm^2^). Nitrided layers in the area of the glow discharge plasma, also with the use of an active screen, have higher values of the current density in the passive range (10^−2^¸10^−1^ mA/cm^2^). The rapid increase in the anode current above *E* = 0.3 V (variants in plasma and initial state) causes the destruction of the passive layer due to adsorption of chloride ions and nucleation of sources of pitting corrosion. Nitrogen increases the sensitivity of the produced surface layers to local corrosion in processes with the use of an active screen under glow discharge conditions (Figure 10).

## 4. Test Results Analysis

The use of an active screen increases the intensity of the nitriding process and increases its temperature. The use of an active screen primarily changes the voltage characteristics, both quantitative and qualitative. Additional voltage pulses arise under the active screen. The values of these voltages are several times higher compared to the voltages occurring during cathodic nitriding and in the potential of the plasma without the shield. The timing of these voltage pulses causes the ions and other active components of the plasma to reach high velocity. The active components of the plasma are implanted into the substrate material, creating a non-equilibrium zone saturated with nitrogen in the top layer, which facilitates nitrogen diffusion into the substrate. These factors determine that in the nitriding process, only in the plasma potential, only the austenite diffusion zone supersaturated with nitrogen is created in the surface layer of austenitic steel. The reason for this layer structure is the low ion energy for the given process conditions. Moreover, these ions are characterized by a negative polarity towards plasma—approx. 20 V. Hence the small effect of the atomization phenomenon. The low energy of the ions is also not enough to precipitate metal atoms from the substrate, which would react with nitrogen atoms to form nitrides. For such conditions, only the phenomenon of nitrogen ions adsorption occurs, which, by diffusing into the iron crystal lattice, forms a solid solution of austenite supersaturated with nitrogen γ_N_. The use of an active screen promotes the formation of additional zones of Crn and Cr_2_N nitrides. To sum up, it should be stated that in the area of the glow discharge plasma, low-energy ions interact with the substrate surface. Therefore, in this area, the phenomenon of knocking out metal atoms from the nitrided substrate does not occur. Only nitrogen ion adsorption and recombination were found. The nitrogen atoms adsorbed on the surface diffuse into the base material. The active screen increases the concentration of nitrogen ions and increases their velocity. Nitrogen ions are implanted in the nitrided surface layer. Additionally, the active screen increases the efficiency of nitrogen mass transport into the nitride substrate. In addition to the phenomena characteristic of cathodic nitriding, the knocking out of nitrides and metal atoms from the surface of the active screen occurs. Nitrides and metal atoms in the space under the active screen—adsorb to the crystal lattice of the nitrided element, facilitating the nitriding process. The presence of nitrides in the space under the active screen and their subsequent sputtering intensifies the process of nitriding of the element placed in the area of the glow discharge plasma.

## 5. Conclusions

The article presents the results of ion nitriding of 316L austenitic steel. Two variants of nitriding were analyzed: in the plasma potential—on the substrate insulated both from the anode and from the cathode, and in the plasma potential with the use of an additional active screen.

The tests were conducted in the temperature range of 430–490 °C at a pressure of 150Pa; the composition of the reactive mixture was as follows: 75%H_2_ + 25%N_2_. The duration of the nitriding process ranged from 5 to 17 h.

The obtained results enabled us to conclude that the use of an active screen in the nitriding process significantly affects the depth of nitrogen diffusion in relation to nitriding only in the plasma potential. The developed models of the structure of nitrided layers on the 316L steel substrate made it possible to determine the characteristic feature of these layers, which is the zone structure composed of CrN and Cr_2_N nitrides and γ_N_ phase grains. The total depth of the nitrided layers when using an active screen changes mainly as a result of increasing the depth of the Cr_2_N nitride precipitation zone. Due to the lower concentration of chromium atoms on the surface, the formation of a nitride zone is limited.

The layers after glow discharge nitriding in the plasma potential have a lower hardness than the layers produced under the conditions of the nitriding process with the use of an active screen. At the same time, the smallest increase in wear resistance was observed for the nitrided layers in the glow discharge plasma potential (especially for a short time and at a low process temperature). In terms of corrosion resistance, the nitriding of the austenitic 316L steel in the area of the glow discharge plasma leads to an increase in the resistance of the nitrided layer to pitting corrosion, with a simultaneous deterioration of the stability of the passive layer.

## Figures and Tables

**Table 1:** Chemical composition of 316L austenitic steel, % wt.

C	Cr	Ni	Mn	Mo	N	Si	P	S	Cu	Fe
0.02	16.82	10.07	1.58	2.07	0.06	0.52	0.028	0.029	0.88	the rest

**Table 2:** Conditions for the nitriding process of austenitic 316L steel.

Process	Temperature, °C	Time, h	Pressure, Pa	Chemical Composition of the Atmosphere
1	490	14	150	H_2_ 75% +N_2_ 25%
2	490	8		
3	460	5		
4	430	8		
5	430	14		
6	460	17		
7, 8, 9	460	11		

**Table 3:** The depth of nitrided layers on the 316L steel substrate depending on the location of the samples in the glow chamber.

Time	Plasma Potential			Plasma Potential + Active Screen			Increasing the Depth of the Nitrided Layer *		
h	Layer depth, µm						%		
	Temperature, °C								
	430	460	490	430	460	490	430	460	490
5	1.3	1.9	2.4	13.6	17.9	18.7	946	842	679
8	2.3	-	3.6	21.3	-	24.9	826	-	592
11	-	3.6	-	-	38.1	-	-	958	-
14	3.9	-	5.5	43.5	-	57	1015	-	936
17	5.1	6.3	6.8	55.1	59.7	66.2	980	848	874

* in relation to nitriding without active screen.

**Table 4:** The depth of nitrogen diffusion and the Cr:N concentration ratio after various variants of glow discharge nitriding.

Process ^(1)^	Nitrogen Diffusion Depth, μm	Concentration Ratio Cr:N on the Layer Surface	Concentration Ratio Cr:N in the Top Layer
P	3.6	13.0:11.9 (1.1:1)	15.0:10.0 (1.5:1)
P + E	38.1	14.2:15.7 (0.9:1)	14.5:7.5 (1.9:1)

^(1)^ P—in the plasma potential, P + E—in the plasma potential with an active screen

**Table 5:** Hardness of nitrided layers on the 316L steel substrate depending on the position of the samples in the glow chamber.

Time	Plasma Potential			Plasma Potential + Active Screen			Increase in the Hardness of the Nitrided Layer *		
h	Hardness, HK0.05						%		
	Temperature, °C								
	430	460	490	430	460	490	430	460	490
5	-	460	-	-	806	-	-	75	-
8	442	-	457	838	-	831	90	-	82
11	-	459	-	-	884	-	-	93	-
14	535	-	578	987	-	982	84	-	70
17	-	663	-	-	1043	-	-	57	-
Hardness in the initial state 218 HK0.05									

* in relation to nitriding without active screen.

**Table 6:** Mass loss of the nitrided layer on the 316-steel substrate for different areas of the glow discharge.

Time	Plasma Potential			Plasma Potential + Active SCREEN		
h	Mass loss, μm					
	Temperature, °C					
	430	460	490	430	460	490
5	-	154	-	-	1.32	-
8	123	-	139	1.1	-	0.95
11	-	128	-	-	0.74	-
14	110	-	83	0.51	-	0.69
17	-	81	-	-	0.34	-

## Data Availability

The data underlying this article will be shared on reasonable request from the corresponding author.
